# The first Phuket International Symposium on Colorectal Disease: postgraduate course of the Asia Pacific Federation Congress 2013 of the International College of Surgeons

**DOI:** 10.1007/s10151-014-1125-0

**Published:** 2014-02-07

**Authors:** E. Silva, M. Boutros

**Affiliations:** 1 Department of Colorectal Surgery, Cleveland Clinic Florida, 2950 Cleveland Clinic Blvd, Weston, FL 33331 USA; 2 Department of Colorectal Surgery, Sir Mortimer B. Davis-Jewish General Hospital, 3755 Côte-Sainte-Catherine Road-Montreal, Quebec, Canada

The first Biennial International Symposium on Colorectal Disease held in Phuket, Thailand, from Dec 8 to Dec 10, 2013, was hosted by the governor of the International College of Surgeons, assistant professor and chief of the colorectal department of the Bangkok Hospital Phuket, Dr. Art Hiranyakas on behalf of the International College of Surgeons, the Society of Colon and Rectal surgeons of Thailand, Vachira Phuket Hospital and Bangkok Hospital Phuket. It was undoubtedly a unique opportunity to hear from internationally acclaimed speakers and experience the most exotic and exceptional Phuket beauty. The three-day exhilarating meeting agenda focused on rectal cancer treatment and advances in colorectal surgery. On the first day, a stimulating live surgery session, moderated by Dr Giovanni Milito, Professor of Surgery, Tor Vergata University Hospital, Rome, Italy, was held showcasing parallel laparoscopic and robotic low anterior resections for distal rectal cancer performed by Drs. Steven Wexner and Seon-Hahn Kim, respectively. The cases were followed by a live demonstration of total mesorectal excision specimen examination and an assessment of mesorectal integrity by the expert gastrointestinal pathologist Dr. Mariana Berho, Chairman of the Pathology and Laboratory Medicine, Cleveland Clinic Florida. This lively and highly informative session prompted an excellent exchange among experts and the attendees.

The Honorary Lecture on “Colorectal Surgical Education—A Prescription for the Future” by Steven Wexner inaugurated the second day of the conference with a comprehensive review of colorectal surgery education and standards of training, followed by an eye-opening exposition of the challenges and goals for future colorectal training around the world. Dr. Steven Wexner’s lecture served as a perfect prelude to the remainder of the day, which highlighted the complex expertise required for state-of-the-art rectal cancer treatment. The multidisciplinary nature of successful rectal cancer was elegantly taken into account throughout the entire meeting with sessions that discussed the complex role of diet in colorectal carcinogenesis, minimally invasive options and outcomes for rectal cancer surgery, radiologic preoperative rectal cancer staging, and pathological staging after neoadjuvant therapy. A sober discussion on sphincter preservation by Dr. Hiranyakas topped the session with a well-balanced review of the oncologic and functional outcomes, highlighting the advantages and disadvantages of this approach. The controversial topic of the benefits of robotic compared to laparoscopic low anterior resection for rectal cancer was presented by Dr. Francis Seow-Choen and was followed by an edifying and interesting debate with excellent audience participation. The challenges of advanced rectal cancer management were also thoroughly discussed including presentations that reviewed the most recent trials and outcomes for the management of stage IV disease by Yik-Hong Ho, cytoreductive surgery and HIPEC for carcinomatosis peritonei and the liver-first approach for metastatic rectal cancer.

The program also included a wide-ranging overview of the diagnosis and treatments of frequent anorectal disorders including pelvic floor disorders, fecal incontinence, and complex anal fistulas. These sessions were coupled with a hands-on endoanal ultrasound course taught by Art Hiranyakas and a live LIFT procedure demonstration by Arun Rojanasakul, Chulalongkorn University, Thailand, who initially developed and reported the procedure. Hemorrhoidectomy techniques and indications followed high-yield topics including management options for complex anorectal fistulas and fecal incontinence. The third day of the meeting also included updates on a potpourri of common anorectal and colorectal issues, including rectal trauma, management of anastomotic leak, and an excellent presentation on enhanced recovery after colorectal surgery protocols and updates, presented by Drs. Varut Lohsiriwat, Mahidol University, Thailand, and Fabio Potenti, Cleveland Clinic Florida, respectively. In addition, a poster walk-around session displayed scientific research abstracts from Asia, Europe, and North and South America.

**Fig. 1:**
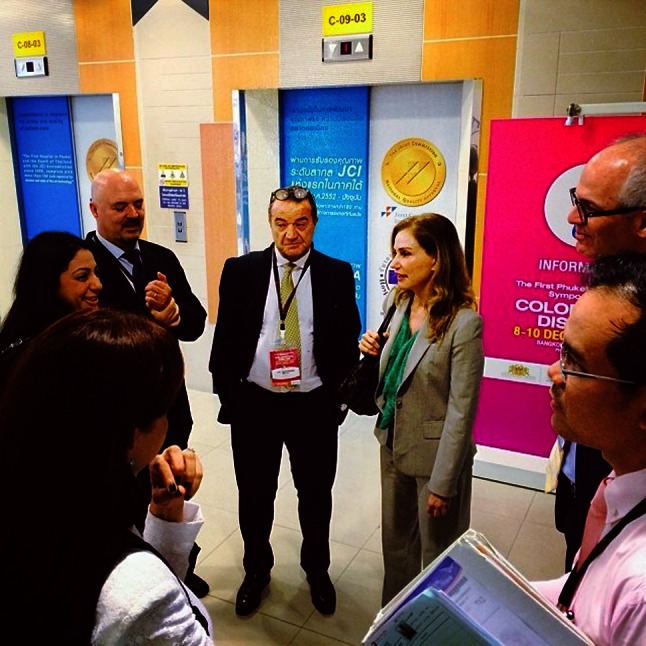
From *right* to *left* Drs. Hiranyakas, Wexner, Berho, Milito, Potenti, Boutros and Silva

The meeting attendance surpassed expectations and absolutely fulfilled the aim of providing an in-depth and stimulating scientific update of rectal cancer management and advances in colorectal surgery. The world-class exchange of knowledge and experience from international leaders in the field of colorectal diseases and live demonstrations made this an outstanding educational meeting. Taken together, with the fascinating natural beauty of Phuket during the most beautiful time of the year, and the finest Thai hospitality, we are all certainly looking forward to participating in the 2015 meeting. Anyone who did not attend in 2013 should indelibly mark the dates of December 9–11, 2015, in their calendar. Anyone with an interest in attending a highly informative meeting and in visiting one of the most beautiful areas in the world should plan to attend. The topics for the next meeting include multidisciplinary approach to colorectal cancer, colorectal imaging, histology, genetics and molecular biology, minimally invasive colorectal surgery including robotics and laparoscopy. Controversies in the management of rectal prolapse, advanced surgical management of fecal incontinence, constipation, anorectal fistulas, and hemorrhoids are also a part of the agenda which includes live surgery and hands-on workshops in surgery, imaging, and histology as well. Save the date! (Fig. 1).

